# Diversity, Complexity and Ordinality: Mental Health Services Outside the Institutions—Service Users’ and Professionals’ Experience-Based Practices and Knowledges, and New Public Management

**DOI:** 10.3390/ijerph18137075

**Published:** 2021-07-02

**Authors:** Alain Topor, David Matscheck

**Affiliations:** 1 Department of Mental Health, University of Agder, 4630 Kristiansand, Norway; alain.topor@socarb.su.se; 2 Department of Social Work, Stockholm University, 106 91 Stockholm, Sweden

**Keywords:** support in daily living, experience-based practice, mental health, social services, new public management, helpful professionals

## Abstract

In conjunction with the dismantling of psychiatric hospitals, social workers have been commissioned to help service users in their daily living in their homes and in the community. The consequences of these changes for experience-based knowledge and practices in their contexts remain relatively unknown. In this study, eighteen service users and the social workers they described as helpful for them were interviewed. The interviews were recorded, transcribed, and analyzed using Thematic Analysis. The following themes emerged: “Here, there and everywhere”, “Doing, being, becoming”, “Talking” and “Order, planning and improvisation” concerning the contradictions service users and professionals mentioned about their practices and the conditions imposed by managerial methods connected to New Public Management. Finally, “Spontaneous planned complexity” was chosen as our overarching theme to characterize the new knowledge and practices which have been developed. The displacement of the place for the encounter and the introduction of non-medicalized professions have allowed community-based practices and thus the co-creation and emergence of new knowledge about the service users as persons and the professionals as qualified professionals. The challenge remains for managers to have trust in their colleagues and not impose rigid rules, schematized methods, and repeated controls.

## 1. Introduction

In the Nordic countries, the overall aim of the dismantling of the mental hospitals was normalization. However, it was not the patients and service users who were to become “normal”, but rather their living conditions [1]: people should have their own apartments, a basic income and possibilities to participate in various social activities. As it was the responsibility of the welfare state to secure acceptable housing, economic conditions and health care for all citizens, persons with mental health problems were included in the social service’s mission, separate from psychiatric services whose aim it is to concentrate on medical and psychotherapeutic interventions.

Thus, in Sweden, a separation was established between the health care and social service sectors. The Psychiatric Health Care Reform of 1995 clarified and cemented this separation. Psychiatric service remained exclusively part of the health care sector, organized on a regional basis and concentrating on a medical treatment model, while social services became the responsibility of local authorities and included provision of acceptable housing, economic resources, and daytime activity centers [2].

With this system for welfare provision, it became necessary for the social services to develop interventions to help and support persons with mental health problems in their homes and various daily living situations [3]. In this article, we contextualize the practices of social work in the home and in daily living and discuss these practices in the context of New Public Management [4], by which organizational principles have come to dominate the welfare sector in Sweden in the years since the Psychiatric Health Care Reform, as it has in many other countries. 

The Swedish word for this kind of intervention is “boendestöd”, which translates directly as “support in housing”. We use the formulation “support in daily living” (SIDL). In SIDL, support workers are assigned to help the individual develop his or her ability to manage situations which occur in daily life. This work is based in the person’s own home and includes issues such as cleaning, preparing food, paying bills, etc., but can also involve situations outside the home, such as shopping or taking exercise. Or it can involve contacts with other services, such as health care, social insurance or library services. 

The aim of SIDL is therefore not to treat patients, but to provide support. This is a major difference as compared to the medically based model of psychiatric services and has extensive consequences, as psychiatric services have no interventions directed to the social life of the persons they treat. “Home social workers”, whom we will call “support workers”, have neither specialized psychiatric knowledge nor treatment method. Unlike psychiatrists and other professionals working in psychiatric services, the locus of their work is not their own home ground (the office or clinic), but the home ground of the individual and the public places where the individual needs to carry out errands or participate in activities. Consequently, the support workers’ prestige and symbolic and legal power is considerably less than that of medical professionals [5]. 

Under these circumstances, support workers develop quotidian-based relationships with service users. Thereby, they develop contextualized knowledge and practices concerning individual service users, but also more generally persons with severe mental health problems living in the community. This is in contrast to what medical professionals can learn about the same persons in time-limited encounters in a psychiatric office or ward. 

To study the knowledge and practices of support workers in SIDL, we can turn to the growing field of research on helpful professional relationships. A possible starting point could be Rosenzweig’s paper [6] on common factors, which describes how different forms of psychotherapy can be more or less equally effective, despite differences in method and theoretical basis. Instead, Rosenzweig points to the importance of factors which are common to outcomes in all psychotherapeutic situations, especially the alliance between the patient and the therapist. Research on common factors in psychotherapeutic settings has continued to develop since Rosenzweig [7,8]. Additionally, concerning other professions in the psychiatric field (including alcohol and drug use), there is a growing number of studies about the importance [9,10] and the content of helpful professional relationships, independently of the professional’s formation and orientation [11,12,13]. We also have access to studies that place “helpful relationships” in a context constituted of places, landscapes, and objects [14,15].

However, we still lack knowledge concerning helpful practices which have developed outside traditional settings and health professions. In a previous article, the authors investigated what elements of user involvement can be found in service users’ and support workers’ descriptions of helpful SIDL [16]. In this article, our aim is to focus on how service users and support workers describe what is helpful in their contact. What is done? Where and how? 

New Public Management (NPM) is a collective term which emphasizes managerial control. Middle managers are required to show performance outputs according to standardized procedures that produce measurable and quantifiable results. There is also a tendency to prioritize economic efficiency over qualitative considerations in service [17,18]. Studies of the effects of NPM on social work have highlighted how professionalism and discretion are undermined in, for example, care of elderly [19], families and young people [20] and other areas of the social service [21]. One aspect of NPM is buying and selling of services according to a purchaser–provider system. This system requires an initial assessment of an individual’s need for service, which can result in a decision granting a specific intervention and a service order to a provider. SIDL is usually granted for a period of a year, with extensions as needed. A service provider for SIDL is then required to draw up a formal plan for the intervention, in consultation with the service user.

## 2. Materials and Methods

Most studies of helpful relationships in mental health settings are based on interviews either with service users [11,22] or professionals [23,24]. However, since relationships occur between two or more persons, we wanted to create a design in which we could ask both the service user and the professional, respectively, about their experiences of co-constructing a relationship which they both acknowledged as helpful. 

Our aim was to collect detailed descriptions of how positive experiences were constituted, and therefore we asked to interview service users who had experiences of helpful SIDL. Thus, our focus is on the construction of helpful relationships. Knowledge about non-helpful experiences is important [25] but is not approached in this study.

### 2.1. Recruiting, Sampling and Interviewing

Qualitative interviews were conducted with services users and support workers in community care in three municipalities. The service users received SIDL with a base in their own apartments. There were no criteria for specific diagnosis or institutional history, but to qualify for this form of support, one had to have received psychiatric interventions and been assessed to have a long-time disability. 

Interviews were conducted individually either by one of the current authors or by a third member who was then part of the research team. As our ambition was to interview pairs of service users and support workers, we interviewed the service user first and then asked the service user to recommend a support worker we could interview. 

Service users were recruited for interviews through notices on bulletin boards at meeting places where service users who receive SIDL would be likely to visit, such as the daily activities center and the local library. Information was also available on the municipalities’ websites and distributed by support staff. We asked to be contacted by services users who had received SIDL for at least 6 months as a voluntary intervention (that is, not as a part of coercive care), either by telephone or by email directly to the researchers. 

We were contacted by 18 services users. All were interviewed, resulting in a selection of 12 women and six men, 34 to 73 years of age. 

Not all support workers could be contacted, and three service users recommended the same support workers, who were then interviewed twice. A total of 16 interviews were conducted with 13 support workers, including nine women and four men. Their experience as support workers ranged from several months to nearly 20 years.

Service users were given the choice whether they wished to be interviewed in their homes, in the municipalities’ facilities or at the interviewer’s office. Support workers were interviewed in municipal facilities during ordinary working hours. The interviews followed semi-structured protocols (see Supplementary Materials) and varied in length between 20 and 105 min. The interviews were audio-recorded and transcribed in full.

### 2.2. Analysis

All interviews were read several times by the authors. Using Thematic Analysis [26,27], preliminary themes were constructed bearing in mind Braun and Clarke’s warning that themes are not to be found in the data but should be seen as the result of a creative process between the authors and the interviewees which begins in the recruitment and interview situation. The analysis followed the six steps described by Braun and Clarke [28]. Through a back-and-forth process between emerging themes and interviews and between the authors, themes were formulated, defined and the most illustrative quotes chosen before each theme was definitely formulated and named to reflect their respective content. This process was handled through use of the usual Windows Office programs, without the help of special software for coding.

In the results section, we present some extensive quotes to reflect the context and dynamic around the described situations, which otherwise could disappear when using thematic analysis.

## 3. Results

SIDL differs from most professional interventions through its diversity and thus its *complexity*. Professionals and service users meet in different places and do different things together, sometimes spontaneously and sometimes carefully planned, and they talk about different topics ranging from distress to how to cook potatoes. Diagnosis was hardly mentioned during the interviews, nor the formal education of the support worker. As the relationship sometimes lasted many years, both the individual’s diagnosis and the support worker’s educational level might change. Therefore, another term could be associated with the first two: ordinality.

It’s to break the isolation. If I want to clean, we clean. If I want to, but it’s seldom, we can wash a window. Yes, and there are these valuable conversations, oh, how I look forward to them! (Service user) 

The following themes emerged from the analysis: “Here, there and everywhere”, “Doing, being, becoming”, “Talking” and “Order, planning and improvisations”. Finally, “Spontaneous planned complexity” was chosen as our overarching theme (Table 1).

### 3.1. Here, There and Everywhere

Although the Swedish term for SIDL stresses the person’s home as the place of the intervention, it appears in the interviews also as a base for excursions to the community outside. Even if the first contact happens in the person’s home and different aspects of housework often are the center of attention, with time it is possible to notice a diversification of the tasks which are done and the places where they are performed. As “here” is the home ground of the person in need of help, “there” might be the necessary places related to taking care of a home:

With SIDL generally, it’s hard to describe what you do, because it sounds like you do not do anything more than just go home to them. You’re mostly just there as a kind of social support. However, we usually do errands, because she gets very scared when she’s outside with other people, she does not want to leave her home. Therefore, then I’m there as a support so we can do her usual errands, go to the drug store, shop food…(Support worker)

Thus, “everywhere” is everywhere else visited by the service user together with the support worker. These could sometimes be places which are not necessary for the maintenance of a basic everyday home life, but rather places of shared pleasure. Sometimes it could involve walks or even an excursion far from what might have been included in the formal planning:

On Thursdays we go and swim at a swim hall. Then, we take a sauna and a shower and just feel good. Enjoy life. (Service user)

These pleasures can come out of doing things together which may not have been scheduled, and they seem to contribute to the (re)construction of the service user as a person. Through the collective action, he/she becomes someone more than just the shortcomings and distress that are the formal basis for the intervention. In contrast to many other interventions, SIDL follows the user to different places where his/her life unfolds. SIDL might also contribute to expanding the person’s enabling landscape [29] by introducing new places connected to sociability and pleasures.

### 3.2. Doing, Being, Becoming

“Doing” is at the core of the encounters between the service user and the support worker. These activities can simply be doing something together, sometimes dividing them into parts. In this example, a service user and a support worker describe the same sequence of activity:

He vacuums and I do a quarter of the job, that is of the whole floor…He vacuums everything and scrubs half, and I scrub half. (Service user)

I start with the vacuuming, I vacuum to here and you vacuum the rest when you come home, or I vacuum everything and you can scrub half the floor. Therefore, we have a little negotiation, he likes that. (Support worker)

Other times, the mere presence of the support worker could give the user the energy to carry out her own “doing” in usual home maintenance activities:

Before, when I was out shopping, I could just put down the basket and leave, because I did not know what I needed to get, or I just panicked. Therefore, I needed to have help shopping, too. However, now it seems like it’s enough just to have someone with me. I do not know why, sometimes I think it’s written ’idiot’ or ’psych-case’ on my forehead and I lose my self-confidence. Or sense of self, rather. (Service user)

Most of the service users we interviewed were lonely. One expression of this social isolation was the basic importance of the support worker’s presence:

It’s one thing if you have a friend and go out for coffee or a pizza. However, to go for coffee by yourself, it’s not the same. However, with my support worker it’s fun just to meet. She’s always happy and never quiet, so she talks, and I talk about what has happened in the world and everything. (Service user)

In this case, it was not the doing, but just the presence of the support worker, the “being” together, and the knowledge that s/he will come at a predetermined time that seemed to be an important source of relief and pleasure.

Another aspect that influences the role of presence is time. In many cases, the service user and professional had met regularly for several years:

I’ve had her for nearly 15 years, and it has developed over time …She feels that she can tell me about things and take me in her confidence about how she’s feeling and things that happen in her relationship with her husband, and other things that make her sad. It can calm her and help her to understand things in a different way. (Support worker)

An important aspect of presence is that the service user does not always have to do the planned activities to feel better. Sometimes it is enough just to be together with somebody else, to have a witness who can acknowledge one’s existence just by being there. 

However, the importance of just being there and being there together is difficult to specify and document within formal “interventions”. There is no formal recognition of how the kind of home social work, which SIDL is an example of, can lead to improvements in the life quality and the sense of self for persons with severe mental health problems. There is no language to express this.

As one service user told us:

I want to have the social interaction. I need it, so to speak. However, it winds up being that I get used to my situation and meet very few other people. Therefore, there is a whole world which disappears for me because I cannot, for example, take public transportation and go to town. There are lots of things I cannot do. However, I want us to be able to go to town and I have to act out of the need that I feel today when I feel brave, today we go to town! I want to see more of that time, the life that I have missed for all these years! (Service user)

Later, when we interviewed the person’s support worker, he mentioned an incident that had happened recently. The two of them had managed to take the subway to an appointment with a dentist in town. On their way back: 

She had gotten a temporary filling and then we began to talk about everything. When the subway stopped at the university, she mentioned that she had studied there, so I asked her more about it and thought that, if I keep the conversation going, she will not have time to feel stressed. I do not know if it was that, or if it was that she just had a really good day, but it went really well. I think she was proud of herself afterwards. Therefore, I said to her several times that, “You must be really proud of yourself now”. We got this done, because I know that it was a really big thing for her. To take public transportation is her greatest fear (…). The next day she was really up: “I have to call this person and see if we can do this and that together”. Additionally, it was as if she felt that a whole new world had opened up for her. (Support worker)

The visit to the dentist was planned, but it was difficult to find a parking space in town, so the service user and the support worker discussed how they would get there. The service user decided to try to go by subway. This sequence is important on a practical level: how to get to the dentist. However, it is also important on an empowerment level: the service user proposed to challenge her fears and travel together with the support worker by public transportation. On the way back they passed the subway stop by the university and suddenly she could remember and tell the support worker about a different period in her life, about capabilities and knowledge she had had. It was something that she could be proud of, which the support worker confirmed. A new world opened for her, not only about her past, but also about the possibility to have contact with former friends, a “becoming”. The trip from the home to the dentist had consequences that could not have been planned. In travelling together with the social worker, the woman could tell him stories about another time in her life. It seems that in doing so, she also reminded herself of who she once had been and therefore who she could become. 

The mere presence of the support worker can be an occasion for the service user to begin doing things s/he needs to do or would like to do. This presence can also be an occasion for doing things together, or in some cases for the support worker to do things which one is not capable of doing oneself on a particular day. However, it can also be an occasion for doing things that were not included in the service order or the formal support plan. Doing together opens for moments of mutuality and shared experiences which go beyond the formal roles. The presence of the other and the activities together are transformed in a process of becoming somebody more than just a case, a collection of shortcomings, and a follower of bureaucratic plans.

### 3.3. Talking

Specialized interventions usually focus on what is wrong with the person, and medical or psychotherapeutic interventions are directed either at the causes of the illness or at minimizing symptoms [30]. As we have seen, activities in SIDL can be directed at creating a basis for everyday life and dealing with its changing challenges. However, it is also about something which we could call “pleasurable things” [31].

In SIDL, talking seems to regain one of its basic aims: sharing. A seemingly aimless exchange of thoughts and experiences, expressing things which are going through one’s mind and reflecting one’s personality in the eyes/ears of somebody else to sense how it is reflected by the other as a picture of oneself:

He knows exactly what he needs to do. Therefore, as soon as I get there then “pop”, the start signal, and he’s on it [laughs]. We talk a little while he’s washing the dishes and that. Sometimes he sits down and drinks a cup of tea and relaxes when I come. (Support worker)

Most talking seems to be casual and spontaneous. It is about nothing special and about everything. It is the pleasure of talking with somebody else, to have somebody to talk to. The experience of mutual sharing is not part of any evidence-based intervention and it cannot be systematized in a method:

It’s a conversation with a friend, because with a friend, well, it depends. I know fragmentary bits of [support worker’s] life; she takes up things from her life in relation to what’s happening in my life. Maybe it’s better to compare with coffee breaks. I’m thinking of how it was when I was working, when you would sit and talk during the breaks, and I think it’s a little like that. (Service user)

Talking about problems seems to be focused on practical solutions and exchange of experiences of similar problematic situations, resulting in a de-construction of fixed divisions of human complexity into roles.

Sometimes talking can be the start of activities that exceed everyday routines and plans, and thus are part of a becoming:

He’s incredibly creative and paints unbelievably much. I think I was the one who gave him the suggestion to have an exhibition room so that he can have a spring exhibition. It was just a suggestion, but he embraced the idea. Then, we planned together what he would show. He invited his closest family and some friends he could feel safe with. It’s something we talk about quite a lot, his creativity. He does film, too, animation films. We have a dialogue about who he’s doing it for, and whether the point is that he is going to show the film or just make it for himself. He has very exciting, interesting thinking about his creations. However, especially the exhibition, I really liked his paintings and felt that his family also should see them. Something to show and be proud of. It’s a strength that he has and I think it’s important that he can show it. (Support worker)

In all these different talks, what exists is the sound of one’s own voice and the reaction of somebody else who heard what you said and responded to it, the sound of somebody else’s voice acknowledging you. Through these different talks we might be able to better understand two common “banal” but re-occurring concepts in recovery research. 

The first is the importance of “being heard”. The experience of being heard involves both the presence of another person hearing you, but also this person acknowledging you through his/her responses and well-intentioned questions about what you have said: a dialogue. The second is that service users often talk about “to be seen”. Although at first the dialogue may be about one’s just being, it can develop to be about possibilities, about becoming. Service users mention helpful professionals seeing the patient/service user as someone more than a mere diagnosis/illness. The construction of one’s sense of self occurs through the eyes, voice, and actions of the other who is not treating you as (only) an assemblage of shortcomings, traumatic experiences and genetic defects [32,33,34].

### 3.4. Order, Planning and Improvisation

The themes above have included examples of being, doing and talking which could not have been planned, or could not have been planned in detail, and thereby do not easily fit with standardized procedures or show in quantitative results. In addition, statements in the interviews expressed dissatisfaction with the limitations that formal plans, rules and policies put on how SIDL could be used. 

The formal plan for the intervention was seldom brought up spontaneously in the interviews, neither by service users nor support workers. When asked, some service users remembered writing the plan together with the support worker, while others did not remember the plan at all or only vaguely. Some service users actually experienced the plan as a hinderance, as demands that they were not ready to meet:

It requires something extra for me to dare [to go to the shopping center]. There are many puzzle bits that have to fall into place. However, it says in the plan that “we should think about [going to the shopping center]” and it makes me really scared! As it’s something that absolutely has to come from me, not because it says so in a paper or because there’s this plan. All the times, the years, when it said so in the plan […] it never happened. (Service user)

Support workers commented that they often made departures from the plan in order to follow the user’s needs and desires, which often varied from day to day, and some support workers appeared to ignore the plan more or less entirely:

A: She appreciates that I do not always follow a straight line. That I can do other things also, which are fun. Human. Q: What does “human” mean? A: To not always exactly follow the rulebook that someone else has decided. As what I work with, I know what I’m doing. I do not always need someone from behind a desk to do a plan that, this is what it will look like, this is what you will do. (Support worker) 

There were also statements, particularly from users, which had the character of complaints about how rules and regulations limited the flexibility and the scope of the intervention. One such instance was when support workers were no longer allowed to use their cars to drive service users to errands or other activities. Shorter shopping tours which had taken maybe 20 min could take hours. The restricted use of the car was also connected to the restriction of SIDL from social activities, which were classified as a different intervention and subject to a separate procedure of application, assessment of need, formal decision, and service order:

Now I have to use SIDL for cleaning instead. That’s important, so it’s just as well. However, it’s also a social thing to drive away and shop. It’s fun to do something different, instead of just being in the flat all the time. Like the way we did before, when we drove away to [a town] and things like that. However, we cannot do that anymore. (Service user)

While some service users appreciated that there was a special intervention just for social activities, others found the separation of help frustrating. The social aspect of SIDL, the “where”, the “doing together” and the “talking” were limited by what were perceived as rigid and unnecessary rules [5,35].

### 3.5. Spontaneous Planned Complexity

In our results, we have identified the themes: “Here, there and everywhere”, which shows that social work in the home can also be the base for social work in other settings; “Doing, being, becoming”, in which doing together or just the support worker’s presence could help the service user in a process of empowerment and becoming; and “Talking”, in which sharing occurs through casual and spontaneous conversations, being heard and seen. These themes are a part of the becoming and seeing oneself as more than a diagnosis or illness. 

As the overarching theme we have chosen “spontaneous planned complexity”. SIDL is planned according to a service order and a formal intervention plan, and the support worker’s visit comes according to a weekly schedule. Within this framework, we find a spontaneous complexity in which the needs and desires of the service user steer the intervention from one occasion to the next, and in which unplanned being, doing and talking occur in connection with different types of situations.

A discordant note is found in the theme “Order and Planning”, in which service users and support workers often found rules, regulations and formal planning to be hinderances in the spontaneous planned complexity of the home social work carried out in SIDL, even when they are meant as tools for service users’ involvement and empowerment. 

## 4. Discussion

The experience-based knowledge and practices developed by street level professionals together with the persons with severe mental health problems whom they encounter have shown the capacity to help those persons manage an acceptable life outside psychiatric care institutions [36]. Therefore, the knowledge and practices should be appreciated, but, as they are often in contradiction to both the dominant bio-medical paradigm and the application of New Public Management (NPM), they tend to be made invisible or looked down on and their importance is seldom acknowledged.

### 4.1. The Co-Construction of Helping Relationships

Studies about helpful relationships between persons with mental health problems and professionals often focus on the content of the relationship from the perspective of one of the partners in the relationships. They stress the importance of the experience of having been seen, heard and respected, but seldom what these experiences consist of and how they are constructed. Our results confirm the importance of mutuality in the relationship and that these experiences are built upon everyday situations in different situations and contexts where both partners can show their capacities and shortcomings. The co-construction of a helpful relationships seems to be facilitated by the diversity of places where the user and the professional meet, the multitude of tasks they encounter together and their capacity to go beyond the formal planning depending on the specific situation and state. The user is the central person, but the activities of the day are negotiated and not dictated by one partner. The experiences that were shared with us point to the importance in many cases of avoiding the fragmentation of services in specialized tasks spread among different professionals, and instead striving to affirm the complexity of the individual.

Two special aspects of our results should be mentioned, Firstly, the importance of pleasures in the helping relationships. Davidson et al. [31] have already mentioned this aspect, but as it is contradictory to the usual image of mental health problems and how treatment should be conducted, it easily becomes invisible. Secondly, the importance of everydayness and improvisation. The discussions and activities mentioned by our informants are seldom mentioned in textbooks or research about golden standards of treatment interventions. However, these findings confirm earlier studies of helpful relationships, such as Skatvedt [34] and Borg and Davidson [37]. They also can be considered in line with sociological studies [38].

The spontaneous planned complexity of the encounters between the support workers and service users in SIDL can be interpreted as diversity ordinality. These different material conditions for the encounters lead to the possible shifting of positions between the service user and the home social worker depending on the specific situation. A diversity of small things existing beyond or under the radar of the traditional clinical gaze [39]. The message sent by these “small things” mentioned in the interviews can be summarized in the re-occurring references to helpful professionals to see “the service user as an individual, not just a patient” [40] (p. 452) and as more than just a “…number, diagnosis, or set of diagnoses…” [41] (p. 280). The basic aspect is the confirmation of the service user as a “fellow human being” [42] (p. 467) and of being an “equal human being” [43] (p. 162).

Here, the “going beyond” the traditional dichotomy of health and/or illness is implemented at a practical level [12,44,45]. Being there, doing things and even nothing together, and small talking includes shared moments and experiences when the limits which divide health from illness and capacity from incapacity are questioned and transgressed in acts which recreate the service user not only as a mere service user and the professional not as a mere professional [46]. 

One often forgotten aspect of this process is its dialogical moment. Contrary to the vision of professionalism as a neutral, scientific application of evidence-based interventions built on an objective diagnosis [47], dialogue is about exploring situations, experiences, and thoughts together. It is a mutual, reciprocal process in which not only the service user is involved and influenced, but also the professional [46,48]. Crawford et al. [49] use the concept “mutual recovery”, and Brown [50] (p. 830) summarizes different definitions of mutuality in the following way:

…not only is mutuality beneficial, but it involves reciprocal transactions and exchanges, mutual influence and responsiveness, interdependency and a sense of common purpose, exercised in an egalitarian manner.

### 4.2. Home Social Work, Standardisation, and NPM

The defining aspects of home social work do not appear to fit well with the kinds of standardized procedures favored by NPM, which prioritizes schematized interventions, quantifiable results, managerial steering, and purchaser–provider “buy-and-sell” models which merchandise helpful social work. The diversity of home social workers’ interventions may be hindered by such an approach, since diversity, flexibility, complexity and ordinality are difficult to register and measure. We argue that a reciprocal dialogical process which supports the service user’s process of recovery and which we have described as “becoming” is not reproduceable by means of standardization but can only be furthered by encouraging professional discretion on the part of individual social workers. Pleasurable situations have mostly to be co-created and are difficult to reproduce in the planning for groups of service users on the basis of their diagnosis or other criteria. 

NPM has been criticized in official reports which have been published by the Swedish government’s Trust Delegation [51,52]. Nevertheless, we were told in the interviews of trends which are consistent with NPM’s continued dominance in social work steering and management. In some municipalities, support workers were not allowed any longer to work outside the home of the service user. Instead, new “specialized professions” were introduced for social activities and other interventions outside home. These tendencies echo results from earlier research in Sweden on SIDL [53].

National and local authorities stress the importance of service user’s preferences and experience, and of professional expertise in decision making [54]. Both these elements are also included in the construction of evidence-based practices [55]. In Sweden, home-based support is recommended by the National Board of Health and Welfare on the basis of experience-based knowledge, with the comment that there actually is insufficient evidence for the effectiveness of this type of intervention [56].

We argue that the continuing trend towards standardization in accordance with the principles of NPM threatens the use of experience-based knowledge, as is indicated by the official stance that there is a lack of evidence for the effectiveness of home-based interventions such as SIDL. There is a risk for a replication of the quantitative fallacy, which according to Yankelovich, writing in 1972 and as quoted in O’Mahony [57], involves a kind of blindness that follows from a one-sided focus on factors and results which can be measured and quantified.

## 5. Conclusions

This study highlights basic aspects of helpful home social work, such as spontaneous planned complexity and diversity ordinality based on mutuality and reciprocity, which support processes of being and becoming and recovery for the service user.

Through being together in different private and public situations, both parts participate in a process of widening their respective field of experience and thus of their sense of self and their knowledge about mental health problems and recovery [58,59,60]. The practices we have described could also be interpreted through the lens of recovery capital [61] as they strengthen the person’s relationships and personal capital.

The mere description of these practices and their recognition as professional contributions to the recovery process of persons with severe mental health problems could constitute the first step in a new direction in the establishment of recovery-orientated services. It would mean not implementing practices developed in another context, but instead exploring the helpful practices which already exist, but which are made invisible by administrative rules and regulations and a bio-medical paradigm [62].

We are fully conscious of the limited number of persons interviewed in this study and the bias created by our focus on helpful professionals according to service users. However, our results are consistent with many recent studies of helpful professionals in the mental health field in various western countries. Our contribution, the contribution of the persons whose experience-based knowledge we asked about, has its roots in the special and seldom studied context of social work on the service users home ground.

Our results may contribute to a contextual understanding of current knowledge and practices.

## Figures and Tables

**Table 1:** Themes and illustrative quotes for “Spontaneous planned complexity”.

Theme	Illustrative Quotation
Here, there and everywhere	On Thursdays we go and swim at a swim hall. Then, we take a sauna and a shower and just feel good. Enjoy life. (Service user)
Doing, being, becoming	There are lots of things I cannot do. However, I want us to be able to go to town and I have to act out of the need that I feel today when I feel brave, today we go to town! Additionally, I want to see more of that time, the life that I have missed for all these years! (Service user)
Talking	It’s a conversation with a friend, because with a friend, well, it depends. I know fragmentary bits of her life; she takes up things from her life in relation to what’s happening in my life. Maybe it’s better to compare with coffee breaks. I’m thinking of how it was when I was working, when you would sit and talk during the breaks, and I think it’s a little like that. (Service user)
Order, planning and improvisation	She appreciates that I do not always follow a straight line. That I can do other things also, which are fun. Human. (Support worker)

## Data Availability

The data presented in this study are available on request from the corresponding author. The data consist of individual qualitative interviews in Swedish and are not publicly available due to confidentiality.

## Supplementary Materials

The following are available online at https://www.mdpi.com/article/10.3390/ijerph18137075/s1, Supplementary Materials S1: Interview protocol service user; Supplementary Materials S2: Interview protocol support worker.
