# Supplementary material for: Diversity, Complexity and Ordinality: Mental Health Services Outside the Institutions—Service Users’ and Professionals’ Experience-Based Practices and Knowledges, and New Public Management
Source: Int J Environ Res Public Health. 2021 Jul 2;18(13):7075. doi: 10.3390/ijerph18137075 (PMC8297002; doi:10.3390/ijerph18137075)
Supplement: Supplementary file 1 [file ijerph-18-07075-s001.zip › Interview protocol service user.pdf]

Intervjuguide – brukare

Frågor om boendestödet
Hur länge har du haft boendestöd?
Hur många timmar i veckan har du boendestöd?
Hur många gånger i veckan har du boendestöd?
Hur många boendestödjare har du?
Vad skulle du säga är anledningen till att du har boendestöd?

Temana
Tycker du att boendestödet är till hjälp för dig? I så fall, kan du ge något exempel? När inträffade det? Vad utspelades det? Vad handlade det om? Hur gick det till? (Du sa det, vad sa hen då? Vad gjorde hen sedan?)

Det exemplet var något som varit till hjälp – vad var det som var så speciellt bra med det? Hur påverkade det dig?

Är det något i som du tycker inte är till hjälp, eller är hindrande för dig? Kan du ge något exempel? På vilket sätt tycker du att det är hindrande eller inte till hjälp?

Hur skulle du beskriva din relation med boendestödjaren/rna?

Har du någon genomförandeplan? Om ja, har du medverkat i att formulera genomförandeplanen?

Bakgrundsfrågor
Födelseår
Kön

Interview protocol – service user

Questions about support in daily living
How long have you had support in daily living?
How many hours per week do you have support in daily living?
How many times a week do you have support in daily living?
How many support workers do you have?
What would you say is the reason that you have support in daily living?

Themes
Do you think support in daily living has been helpful to you? If you have, can you give an example? When did it happen? What happened? What was it about? How did happen? (What you said, what did he/she say then? What did he/she do then?)

About the example of something that was helpful – what was particularly good about it? How did it influence you?

Is there something that you think has not be helpful, or has been a hinder for you? Can you give an example? In what way do you think that it was a hinder, or not helpful?

How would you describe your relationship with you support worker/s?

Do you have a formal intervention plan? If so, did you participate in formulating the plan?

Background questions
Year of birth
Gender
