# Supplementary material for: Diversity, Complexity and Ordinality: Mental Health Services Outside the Institutions—Service Users’ and Professionals’ Experience-Based Practices and Knowledges, and New Public Management
Source: Int J Environ Res Public Health. 2021 Jul 2;18(13):7075. doi: 10.3390/ijerph18137075 (PMC8297002; doi:10.3390/ijerph18137075)
Supplement: Supplementary file 1 [file ijerph-18-07075-s001.zip › Interview protocol support worker.pdf]

Intervjuguide boendestödjare

X har namngett dig som en boendestödjare som varit till hjälp för hen.

Hur skulle du beskriva den relation du har med X?

Kan du ge något exempel på något du gjort som du tycker varit till hjälp för X? När inträffade det? Var utspelades det? Vad handlade det om? Hur gick det till? (Du sa det, vad sa hen då? Vad gjorde hen sedan?)

På vilket sätt tror du att det varit till hjälp för X? Hur kan man förstå den kopplingen?

Hur förhåller sig det här som du berättat till genomförandeplanen för X, finns det med där?

Bakgrundsfrågor
Födelseår:
Kön:
Utbildning/tidigare yrkeserfarenhet:
Vilket år började du arbeta som boendestödjare?

Interview protocol – support workers

X has given us your name as a supporter who has been helpful to her/him.

How would you describe your relationship with X?

Can you give an example of something you have done which has been helpful to X? When did it happen? Where did it happen? What was it about? How did it happen? (You said what, and what did he/she say then? What did he/she do then?)

In what way do you think you have been helpful to X? In what way can we understand the connection?

Is what you have told us included in the formal intervention plan for X? In what way?

Background questions
Year of birth:
Gender:
Education/previous professional experience:
What year did you begin work as a support worker in daily living?
